# Supplementary material for: Genomic inference of the metabolism and evolution of the archaeal phylum Aigarchaeota
Source: Nat Commun. 2018 Jul 19;9:2832. doi: 10.1038/s41467-018-05284-4 (PMC6053391; doi:10.1038/s41467-018-05284-4)
Supplement: Supplementary file 1 — Supplementary Information [file 41467_2018_5284_MOESM1_ESM.pdf]

Supplementary Information

Genomic inference of the metabolism and evolution of the archaeal phylum Aigarchaeota

Hua *et al.*

Supplementary Notes

Supplementary Note 1: Functional annotation and metabolic reconstruction of genome bins.

Protein coding sequences (CDS) were determined using Prodigal[1]with the "-p single" option for all the genome bins. Ribosomal RNA-coding regions were called using RNAmmer[2]. Functional annotations were conducted based on comparisons with the KEGG[3], arCOG[4] and eggNOG[5] databases using DIAMOND[6] with E-values < 1e-5. Putative carbohydrate-active enzymes were identified based on the comparison to Carbohydrate-Active enZYmes (CAZy) database[7]. The predicted putative endoglucanases in JZ bin_40 and JZ bin_19 were further verified using NCBI's BLAST webserver to identify their conserved domains[8]. To reconstruct the metabolic pathways, CDS for the six genome bins were annotated at the KEGG automatic annotation server (KAAS)[9]. CRISPR regions were checked using CRISPRfinder[10]. Alternatively, the six genomes were also annotated using IMG-M (https://img.jgi.doe.gov/cgi-bin/m/main.cgi).

Carbon, hydrogen and sulfur related metabolic pathways and energy cycling were investigated based on the IMG-M annotation results by extracting corresponding KO and COG from each bin (as listed in Supplementary Table 2). Several genes belonged to complex I of respiratory chains were identified in aigarchaeal genomes, which show close relationship to group 4 membrane-bound NiFe hydrogenases. Previous reported strategy was introduced to distinguish them[11]. Briefly, ScanProsite was used for short motif detection[12]. Matching the *nuoD* homologues with CXXC motifs were classified as Group 4 NiFe hydrogenases, or they were belonged to complex I or energy-converting hydrogenase related complex (Ehr).

Interpro annotation, which has already been integrated into IMG-M, could be used to detect Eukaryotic signature proteins (ESPs) mainly including GTPases, Eukaryotic-specific RNA polymerase, ubiquitin modifier system and ESCRT complex proteins. Besides, arCOGs identified from local comparison to arCOG database were used to detect ESPs. Those conserved domains for proteins related to ubiquitin modifier system were determined use InterProScan online with default parameters[13]. Preliminary models were built with SWISS-MODEL[14].

Supplementary Note 2: Phylogenetic and phylogenomic analyses.

Concatenated ribosomal protein phylogeny. A total of 16 conserved marker genes previously determined to be universally present and rarely horizontally transferred were selected to reconstruct the phylogenomic tree[15]. Marker genes sets were extracted separately using AMPHORA2[16]. Multiple sequence alignments (MSAs) of the individual marker gene sets were built using MUSCLE (iterate 100 times)[17]. Poorly aligned regions were eliminated by TrimAl with the parameters as: -gt 0.05 -cons 50[18]. The phylogenomic tree was reconstructed using RAxML v7.2.7 using the amino acid substitution model JTT, with Gamma rate heterogeneity and invariable sites enabled. Node support was calculated using 100 fast bootstraps[19].

16S rRNA gene phylogenetic analyses. To reconstruct the phylogenetic tree based on 16S rRNA gene, RNAmmer[2] was first used to predict the near-complete 16S rRNA for each genome. The genomes with failure prediction were researched against RDP database[20] using the BLASTn program. The best hits with length < 300 bp were removed for the later phylogenetic analysis. The combined 16S rRNA sequences were then aligned using the SINA alignment algorithm through the SILVA web interface[21,22]. The generated full alignments were filtered to remove those columns comprised of more than 95% gaps. A maximum likelihood phylogenetic tree was inferred by the RAxML (v7.2.7)[19] using the GTR nucleotide substitution model, with gamma rate heterogeneity and invariable sites enabled. The generated tree was visualized by iTOL v3[23].

DsrAB. Reference datasets of *dsrAB* gene (concatenation of *dsrA* and *dsrB* gene) were derived from Müller et al.[24]. CD-hit algorithm[25] was applied to cluster those reference sequences into gene families (options: -c 0.9, -aS 1, -g 1 and -r 1) and representatives for each family were selected for the further tree construction. Alignments were generated using MUSCLE[17] with the default parameters. Phylogeny was conducted using RAxML (v7.2.7)[19] under the JTT + Gamma model of evolution with 100 bootstrap iterations.

CoxL. All coxL gene sequences were selected from King et al.[26,27] and downloaded from the NCBI database. Combine with eight coxL genes detected among the six Aigarchaeota genomes, sequences were aligned using MUSCLE[17] and RAxML (v7.2.7)[19] was used for the inference or phylogeny with the parameters described as above.

Hydrogenases. References of different types of hydrogenases were selected from Greening et al.[28]. MSAs were conducted using ClustalW[29] and Neighbor-Joining tree was generated using MEGA under p-distance model[30]. The final tree was visualized using iTOL[23].

Supplementary Figures

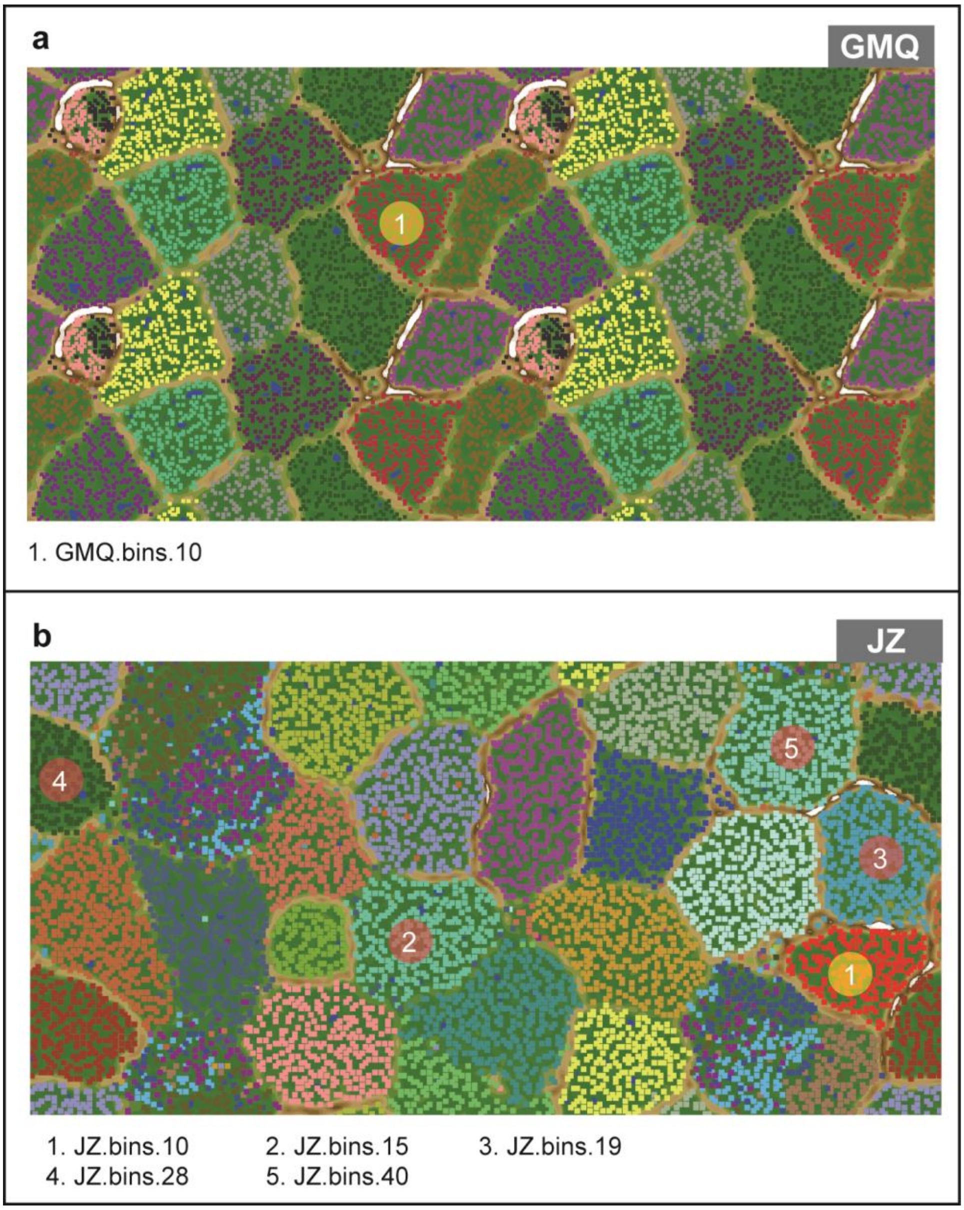

Supplementary Figure 1. Visualization of six aigarchaeal genomic bins in this study using ESOM (Emergent Self-Organizing map)[31]. The sampling sites are: a) GMQ; and b) JZ hot springs in Tengchong in Yunnan Province, China.

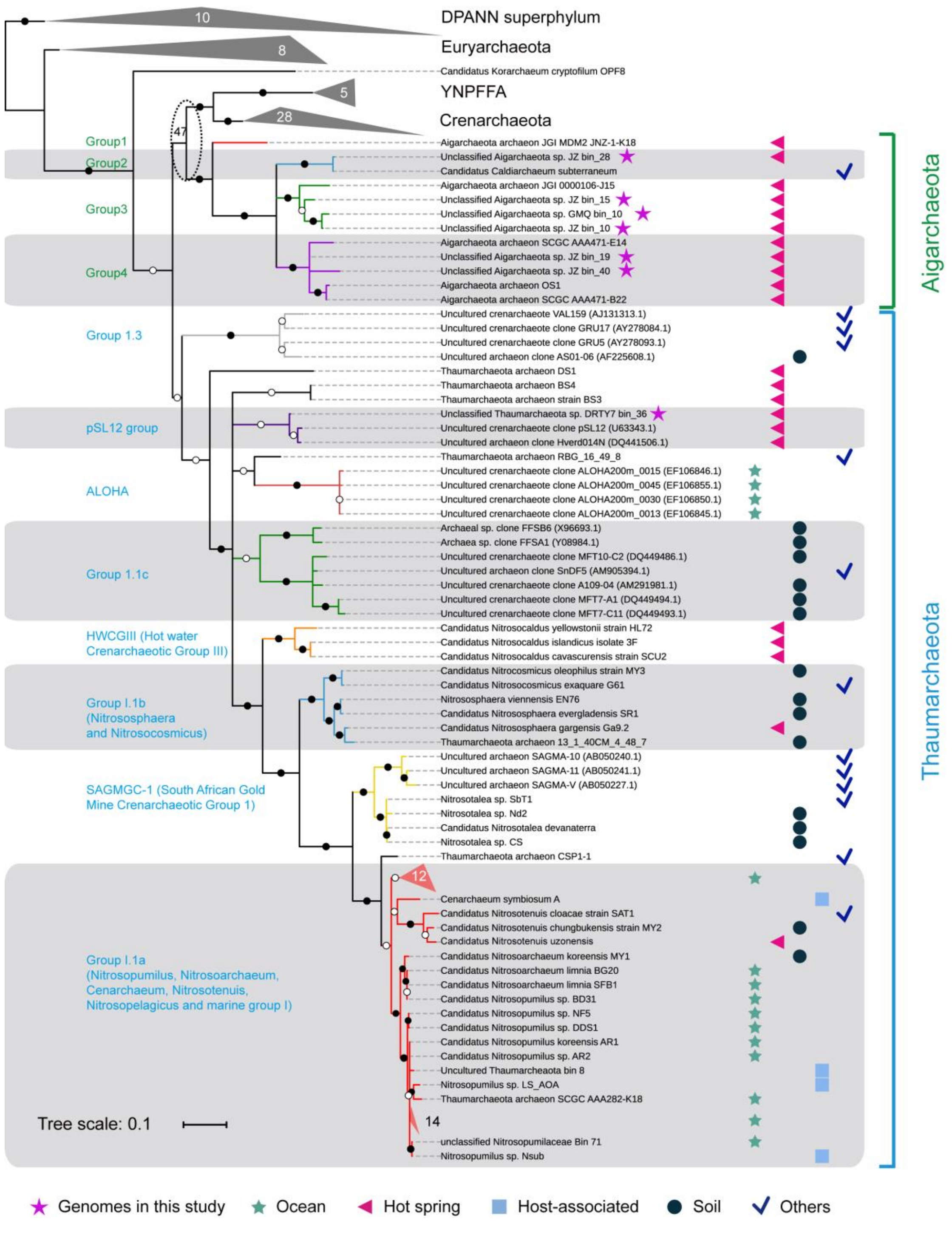

Supplementary Figure 2. Maximum likelihood tree depicting the 16S rRNA gene-based phylogeny of the 67 genomes under Aigarchaeota and Thaumarchaeota. Genomes from the DPANN superphylum were chosen as outgroups. Solid and hollow circles on the branches indicate the bootstrap value ≥ 90 and > 70 respectively. Pink triangles, black circles, green stars, dark blue ticks and light blue rectangles denote the habitats they populated. All the picked 16S rRNA genes were classified into multiple groups based on sequence similarity using CD-hit[25] with a threshold of 94%.

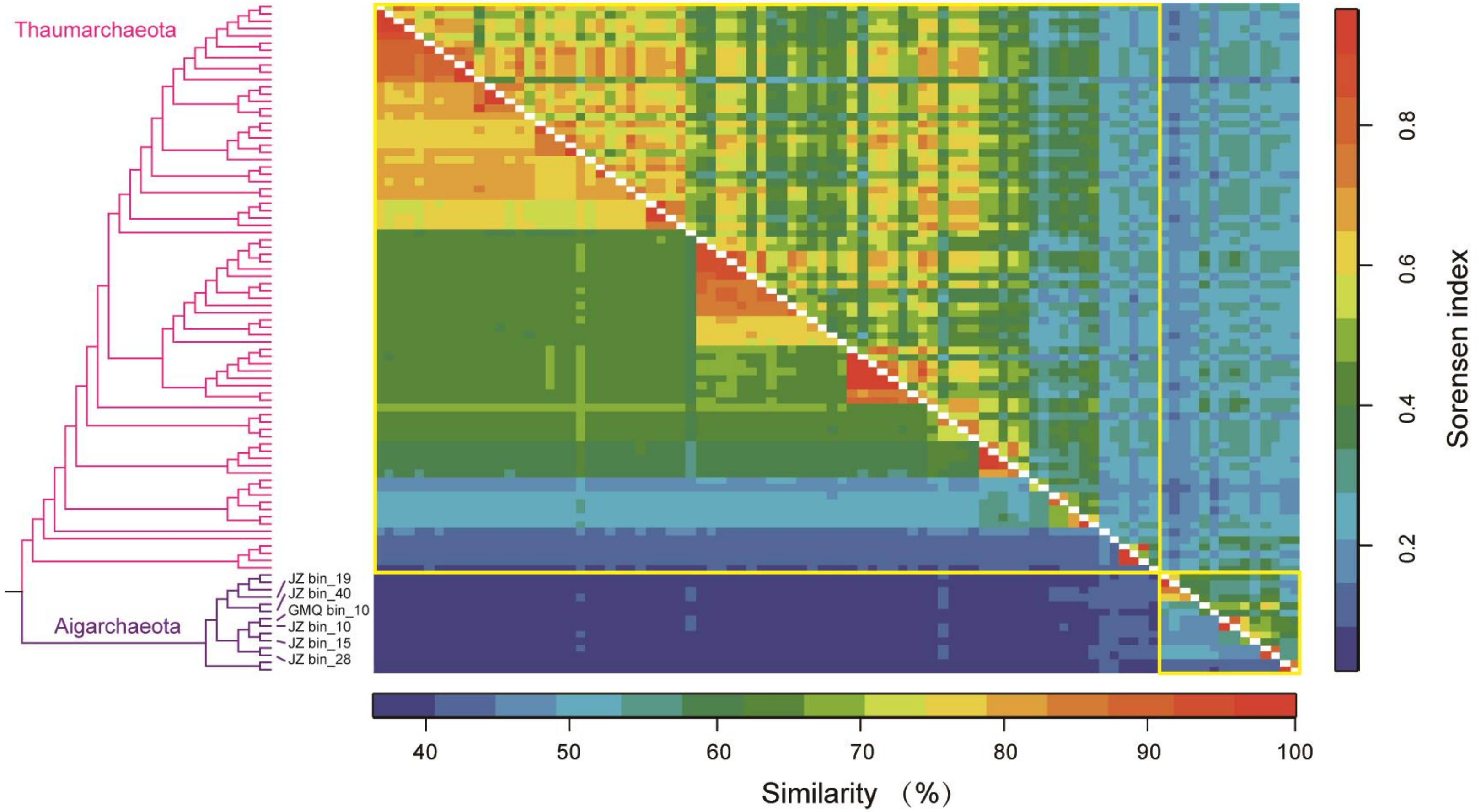

Supplementary Figure 3. Hierarchical clustering heatmap based on average amino acids identity (AAI, lower left of the square) and shared homologues (upper right of the square) for each genome pair. The dendrogram shows the phylogenetic relationships as determined by the maximum likelihood tree analysis of the concatenated alignment of 16 ribosomal proteins retrieved from all genomes. Trees with pink and purple colors show genomes belonged to Thaumarchaeota and Aigarchaeota respectively. Genome self-comparisons are presented in white.

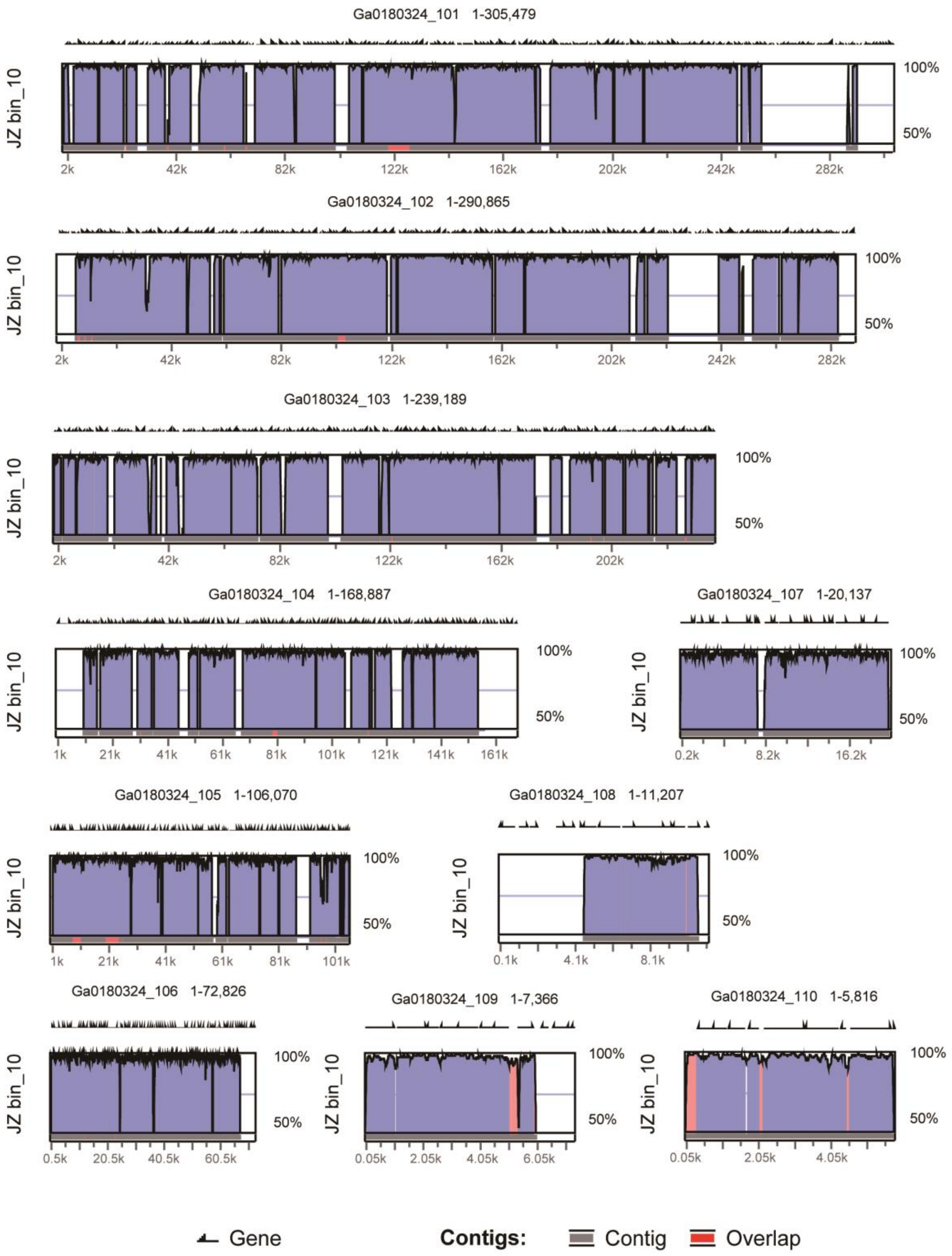

Supplementary Figure 4. Whole genome comparison between GMQ bin_10 and JZ bin_10 using VISTA[32]. The draft genome of GMQ bin_10 has nine scaffolds and was used as the reference. Scaffold names are indicated in panel titles. Black arrows below the scaffold names represent the predicted genes and its orientation in the genome.

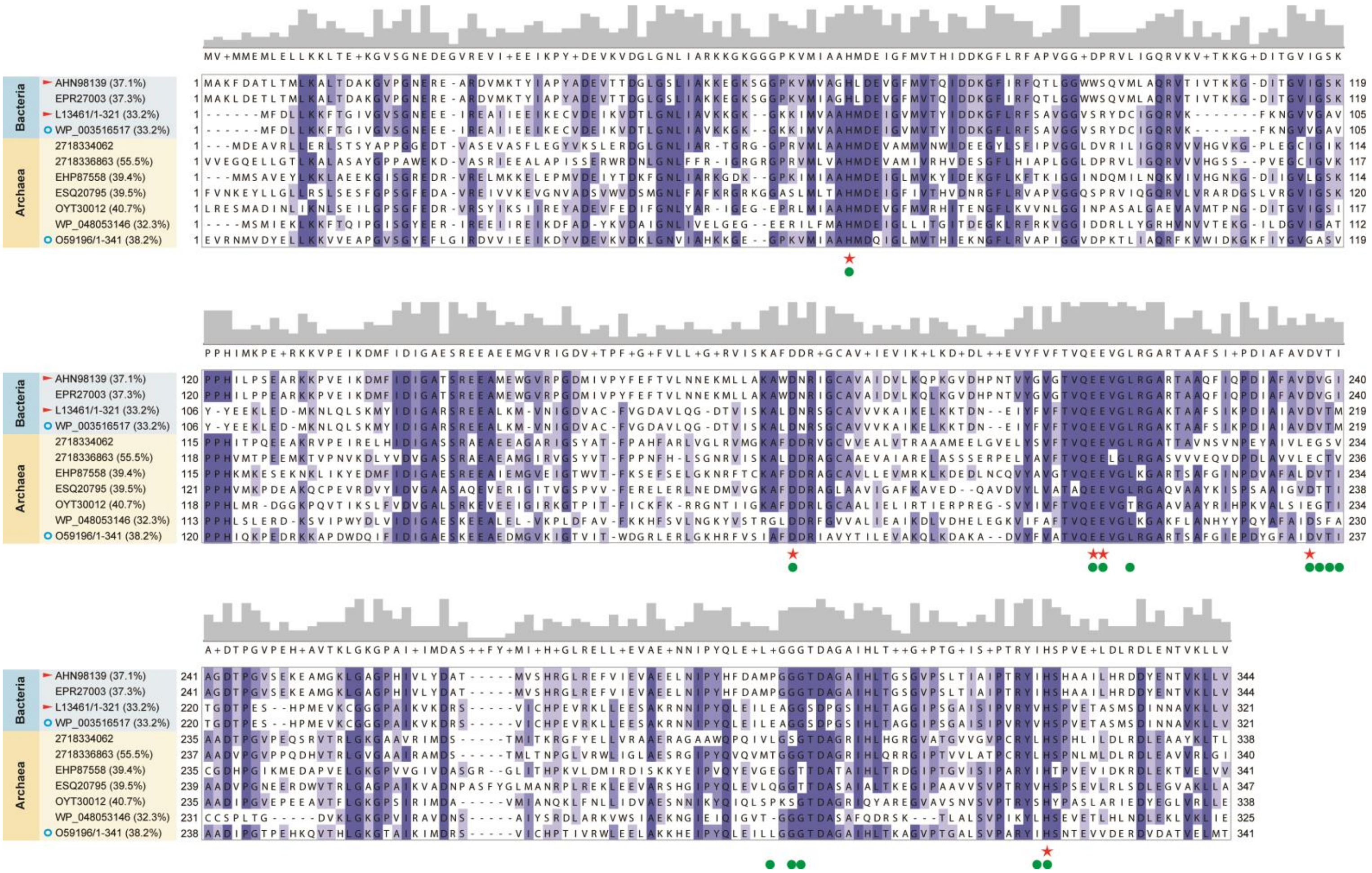

Supplementary Figure 5. Multiple sequence alignments (MSAs) of predicted endoglucanase encoded genes in JZ bin_40 and JZ bin_19 with other verified or possible cellulases. MSA analysis was conducted using ClustalW[29]. The amino acid identity for each gene compared to JZ bin_40 was shown in bracket behind the gene name. Red triangles show the verified cellulase and blue circles show genes predicted as aminopeptidases. The active site residues are highlighted in green circles and metal binding sites are highlighted in red flags. Grey histograms show the consensus amino acids and conservation of each site. The MSAs were visualized by Jalview2[33].

a

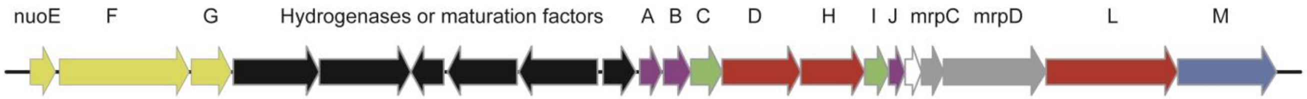

b

Gene_ID	Length [AA]	Pfam ID	E-value	Annotation†	Domain organization‡
2718338905	122	PF01257	2.75E-37	NADH-quinone oxidoreductase subunit E	nuoE: nuoE
2718338906	656	PF01512	0.00E+00	NADH-quinone oxidoreductase subunit F	nuoF: nuoF
2718338907	205	PF13510	1.59E-31	NADH-ubiquinone oxidoreductase-G iron-sulfur binding region	nuoG: nuoG
2718338914	109	PF00507	3.49E-03	NADH dehydrogenase subunit A	nuoA: nuoA
2718338915	146	PF01058	1.84E-46	Ech hydrogenase subunit C	nuoB: nuoB
2718338916	156	PF00329	6.37E-37	NADH:ubiquinone oxidoreductase subunit (chain C)	nuoC: nuoC
2718338917	397	PF00346	1.59E-109	NADH-quinone oxidoreductase subunit D	nuoD: nuoD
2718338918	321	PF00146	2.32E-80	NADH-quinone oxidoreductase subunit H	nuoH: nuoH
2718338919	116	PF13187	1.00E-25	Formate hydrogenlyase subunit 6	nuoI: nuoI
2718338920	81	PF00499	2.58E-03	Protein of unknown function (DUF4040)	nuoJ: nuoJ
2718338922	106	PF00420	3.31E-09	Membrane bound protein complex subunit mbxG	mrpC: oxidored_q2
2718338923	515	PF00361	5.28E-71	Multicomponent Na+:H+ antiporter subunit D	mrpD Proton_antipo_M
2718338924	665	PF00361	8.76E-150	NADH-quinone oxidoreductase subunit L	nuoL: nuoL
2718338925	494	PF00361	1.60E-78	NADH-quinone oxidoreductase subunit M	nuoM: nuoM

c

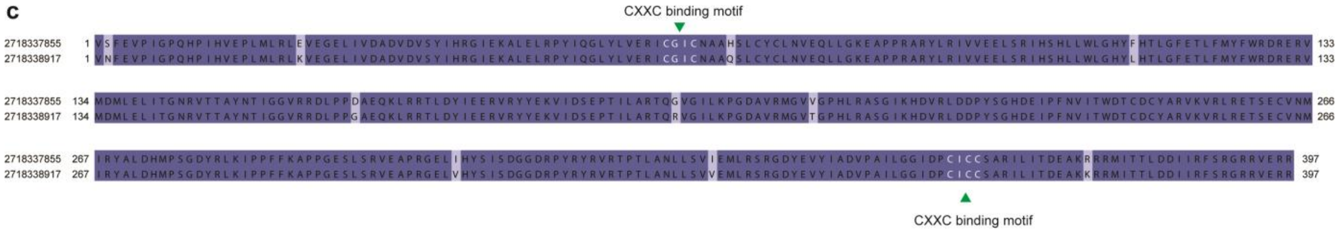

Supplementary Figure 6. Identified NADH ubiquinone oxidoreductases (*nuo*) in GMQ bin_10 and JZ bin_10. (a) Schematic representation of gene cluster organization presents in these two bins. (b) The identification of conserved domains for *nuo* operon. The subunits were color-coded based on the criteria reported previously[11]. †The genomes were uploaded to IMG-M database to conduct the functional annotation. ‡Conserved domains (CD) were identified using CD-search to CD database (CDD)[34]. (3) The identification of CXXC binding motifs in GMQ bin_10 and JZ bin_10 (see Methods for detailed information). The MSAs were conducted using ClustalW[29] and visualized using Jalview2[33].

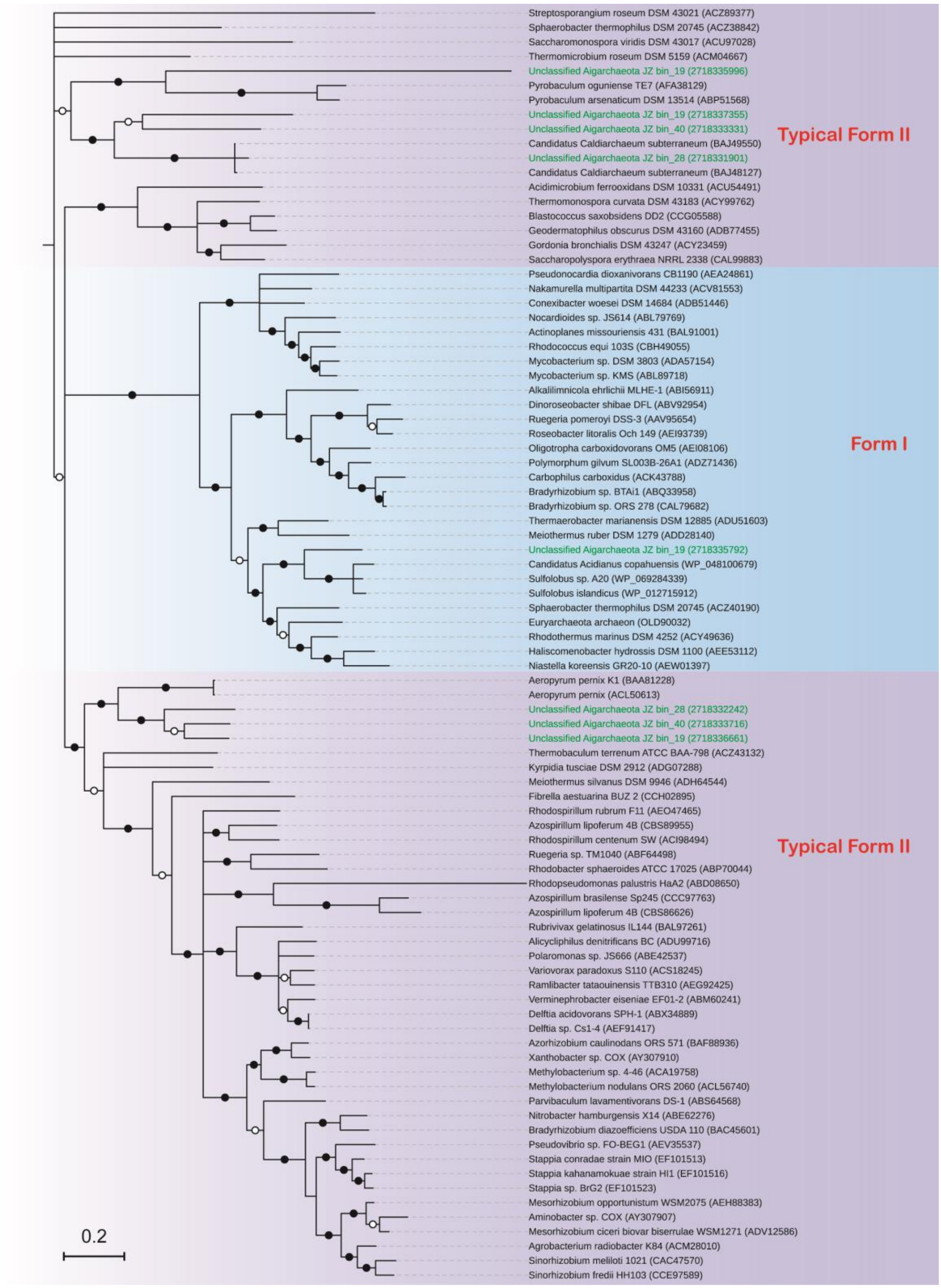

Supplementary Figure 7. Phylogenetic tree of the *coxL* gene. Eight *coxL* genes identified in this study (green taxon labels) were chosen to construct the maximum-likelihood tree. Node labels are the bootstrap support values. Bootstrap support with values > 90 and >70 are shown in solid and hollow circles. Labels in green show the genomes presented in this study. Lineages with blue and purple show the Form I and II type of CODH respectively.

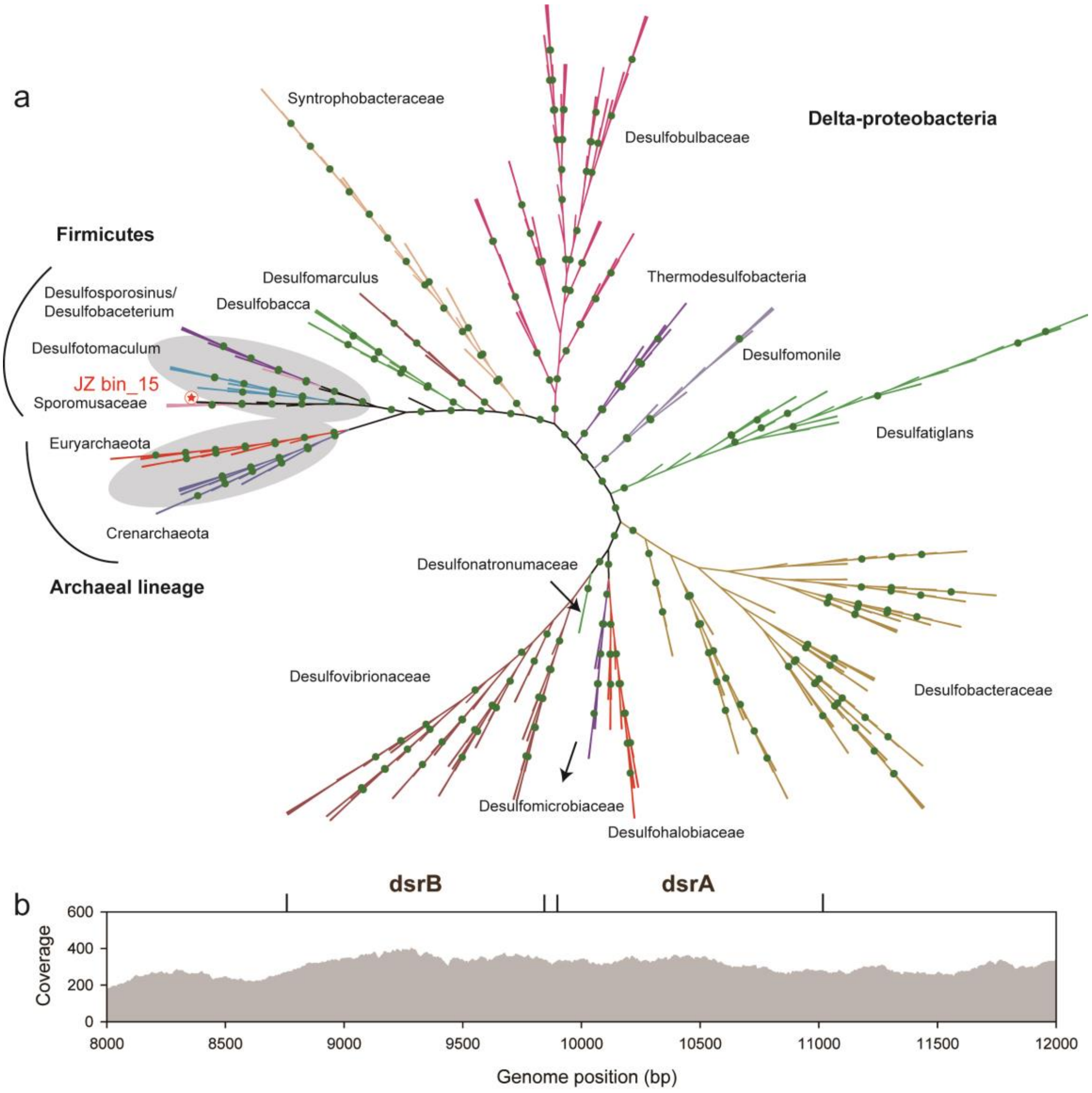

Supplementary Figure 8. Phylogenetic analyses of the *dsrAB* gene in JZ bin_15. (a) Maximum likelihood phylogenetic tree showing the placement of *dsrAB* in JZ bin_15 (red star). Other sequences in the tree were derived from Müller et al.[24]. (b) Histogram showing the coverages of the two genes in the corresponding scaffold. Nodes with bootstrap value ≥ 50 are shown as green circles.

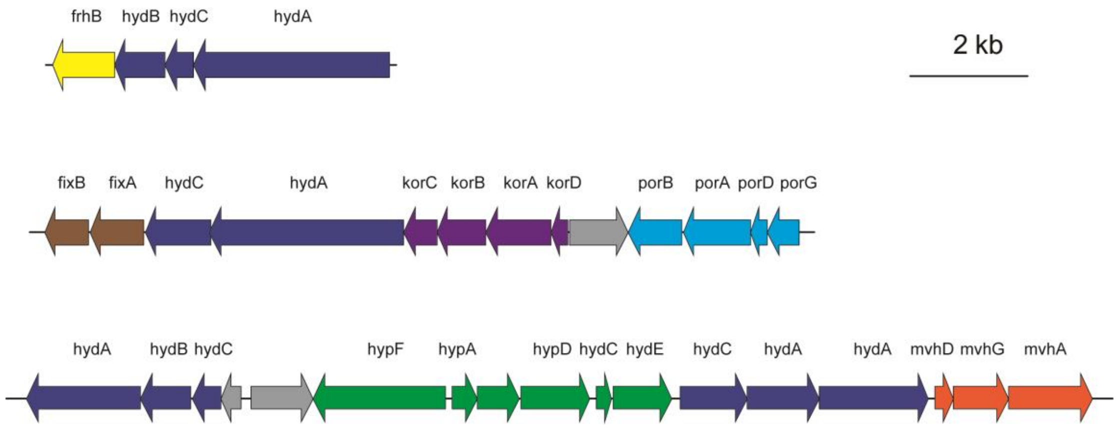

Supplementary Figure 9. Genomic organization of the heterodisulfide reductase (*hdr*) complex and related genes in JZ bin_15. The color code is: yellow for Coenzyme F420-reducing hydrogenase (*frh*), dark blue for heterodisulfide reductases (*hdr*), light blue for pyruvate ferredoxin oxidoreductase (*por*), brown for electron transfer flavoprotein (*fix*), purple for 2-oxoglutarate/2-oxoacid ferredoxin oxidoreductase (*kor*), green for hydrogenase maturation factor (*hyp*), and orange for F420-non-reducing hydrogenase (*mvh*).

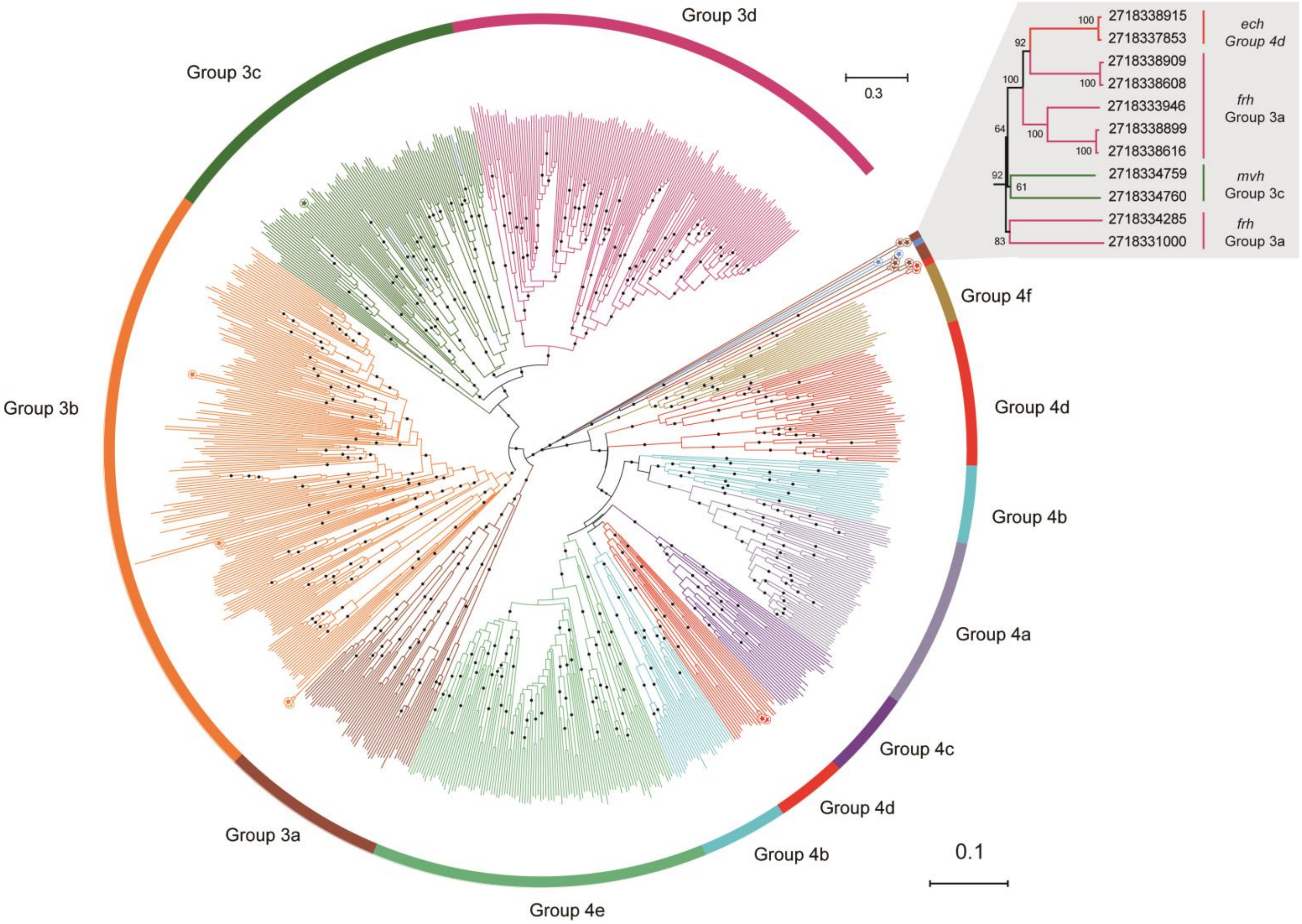

Supplementary Figure 10. Phylogeny of the NiFe hydrogenases. Leaves with a star at the end denote NiFe hydrogenases identified in the aigarchaeal genomes assembled in this study. Reference sequences were derived from Greening et al.[28].

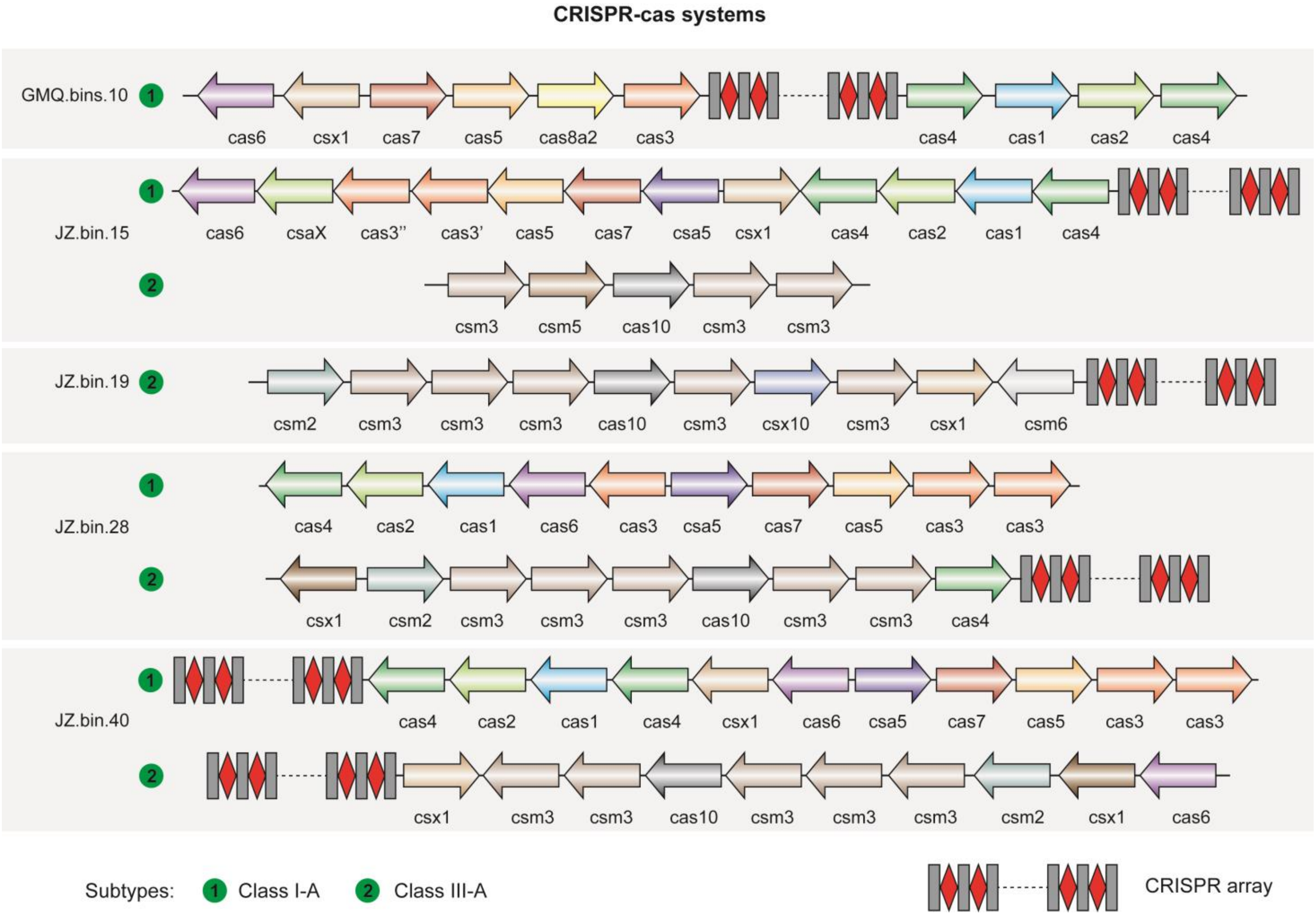

Supplementary Figure 11. Schematic overview of subtypes of CRISPR-cas systems identified in phylum Aigarchaeota. Homologous genes are color-coded and labeled by the family name.

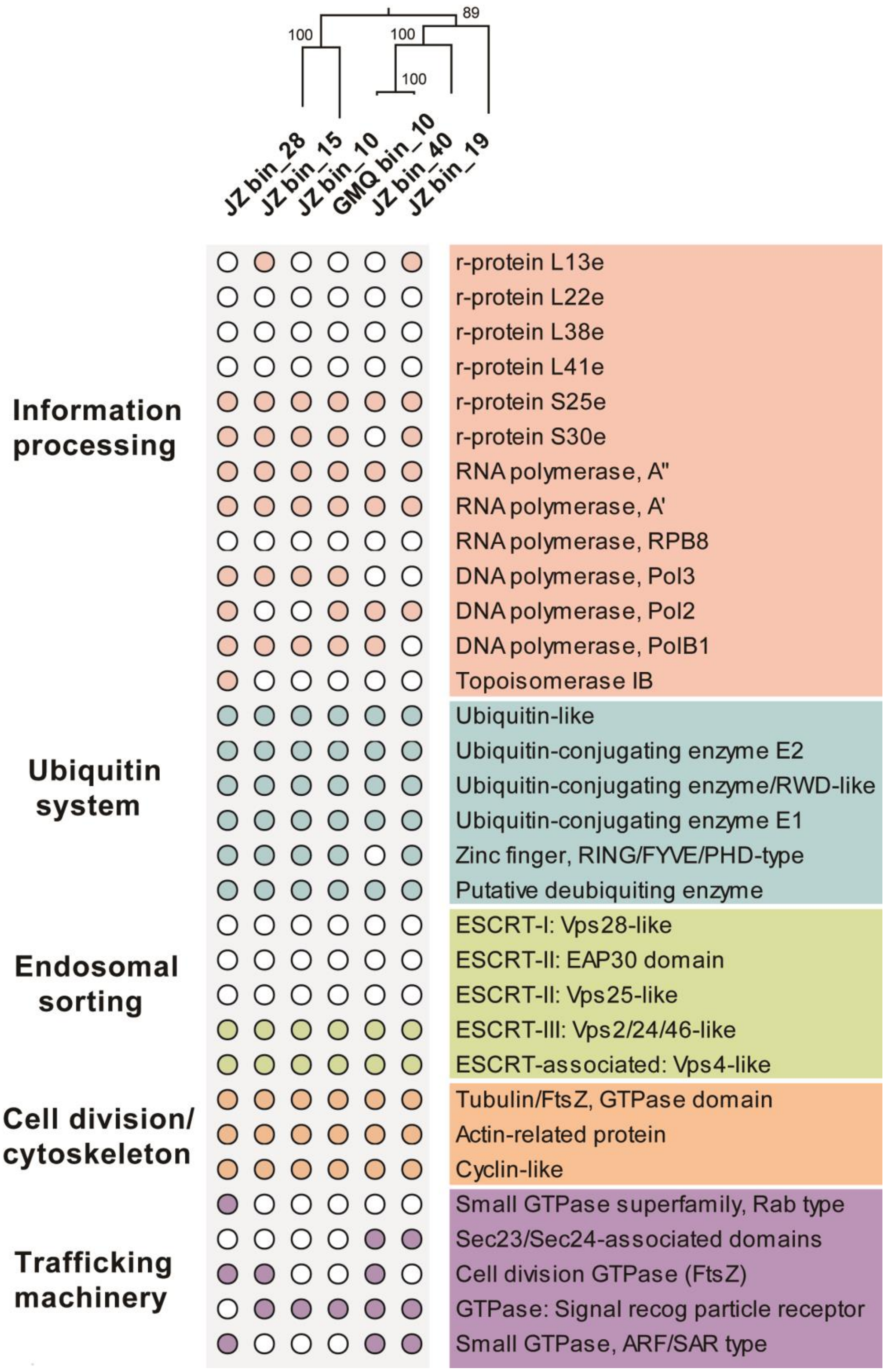

Supplementary Figure 12. Schematic tree of the aigarchaeal genomes reconstructed in this study and corresponding overview of identified eukaryotic signature proteins (ESPs). Solid circles indicate the presence of corresponding ESP.

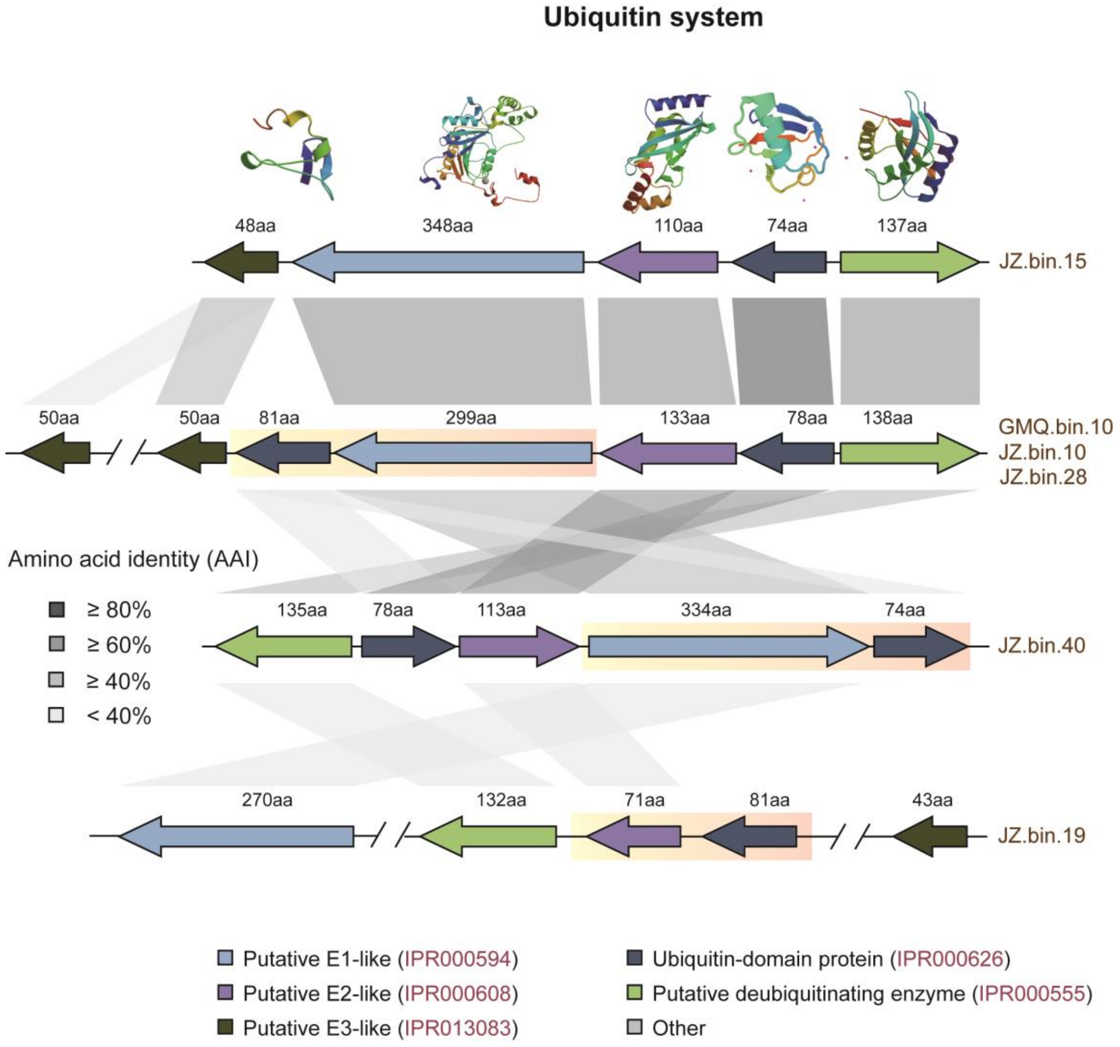

Supplementary Figure 13. Architectures of the genomic loci for the ubiquitin systems in Aigarchaeota. Homologous genes are color-coded and corresponding predicted protein models for JZ bin_15 are shown above. The shaded genes in rectangular with yellow gradient indicate one single ORF containing both of the conserved structural domains. Intensity of the shade connecting two homologous sequences is correlated with pairwise BLAST bit score.

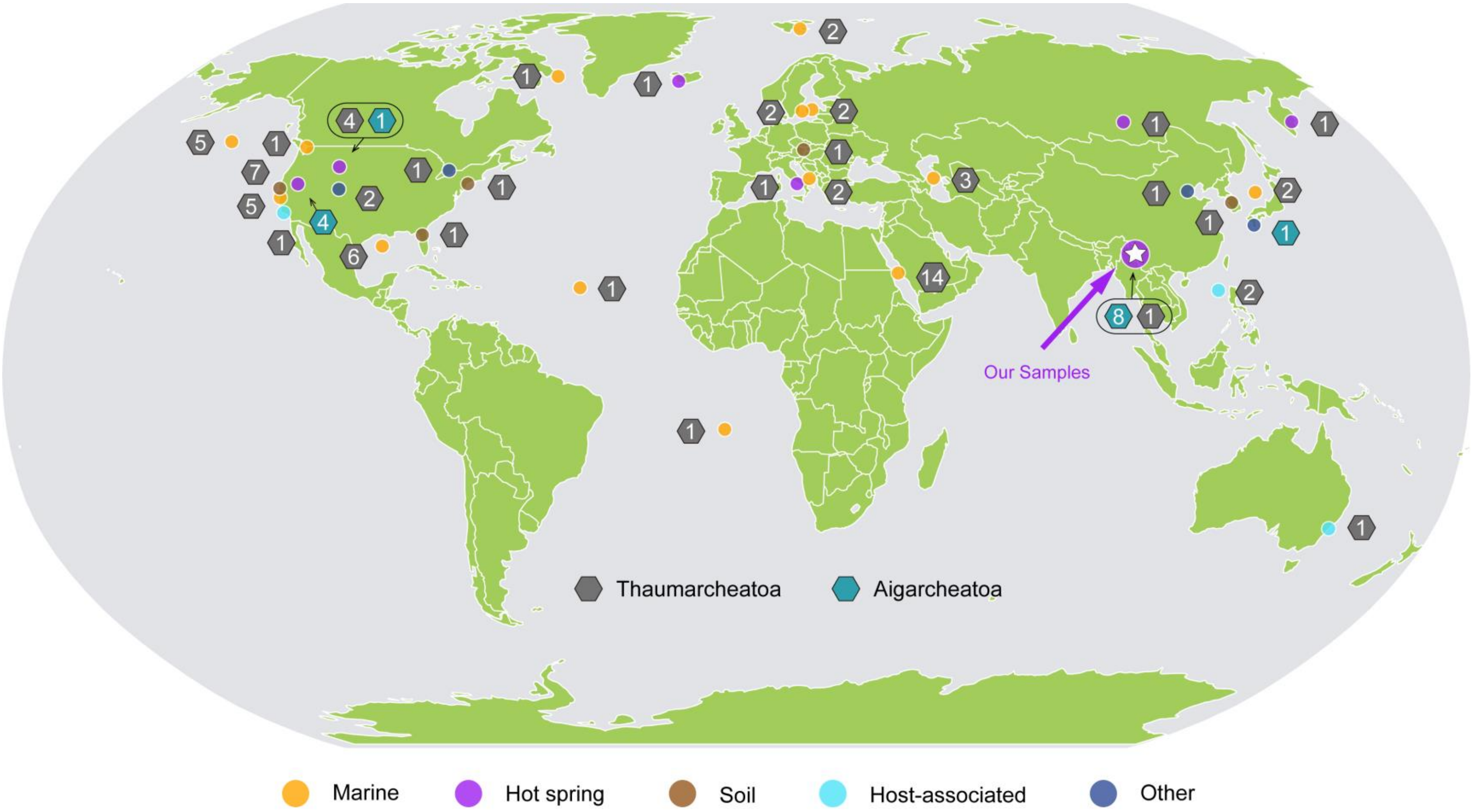

Supplementary Figure 14. The geographical distribution of the sequenced genomes under Aigarchaeota and Thaumarchaeota from the public databases. Refer to Supplementary Table 4 for the information of the genomes and the sampling sites.

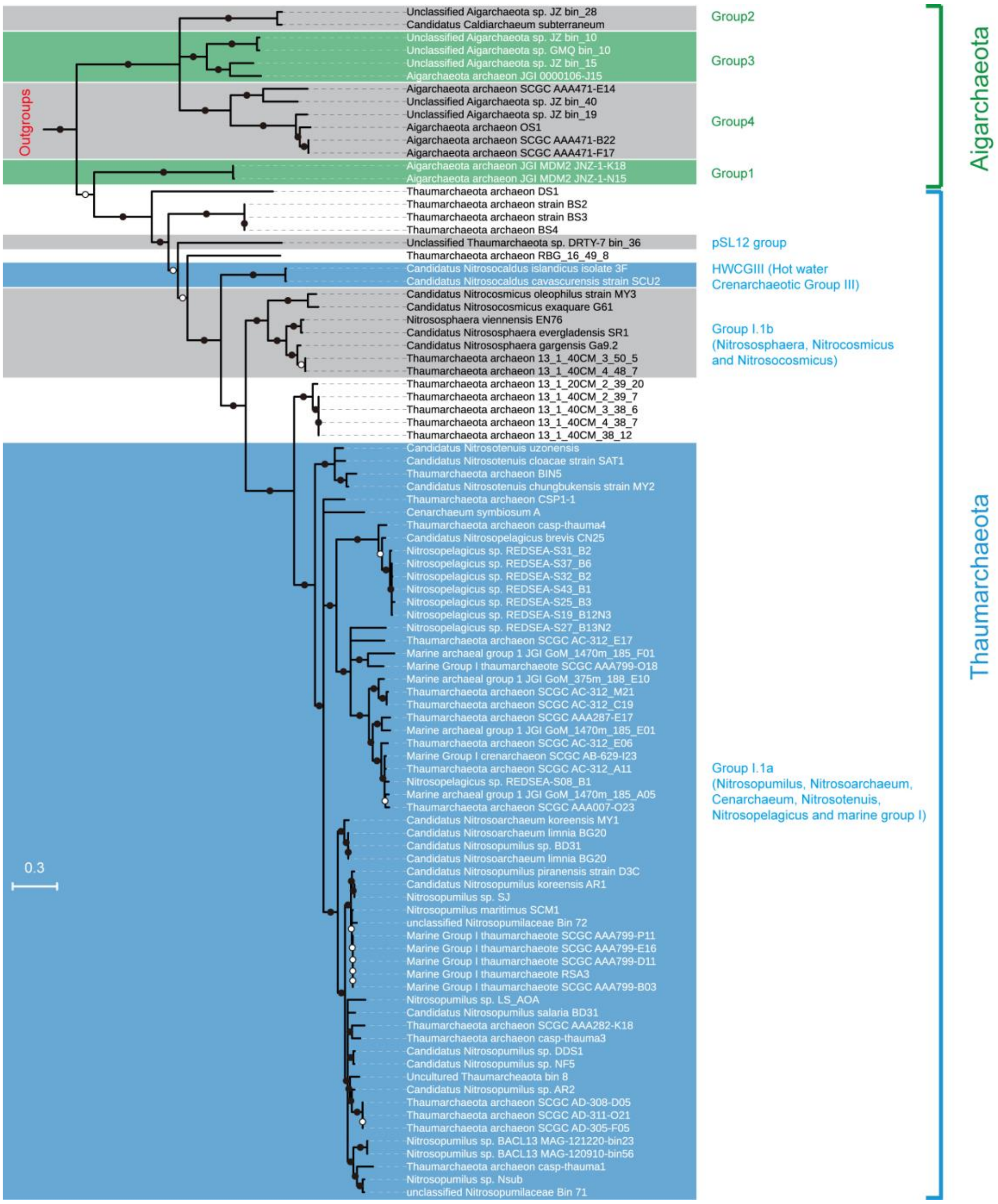

Supplementary Figure 15. Maximum likelihood tree of Aigarchaeota and Thaumarchaeota reconstructed from 10 RNA polymerases including *rpoA1* (K03041), *rpoB* (K13798), *rpoD* (K03047), *rpoF* (K03051), *rpoH* (K03053), *rpoN* (K03058), *rpoK* (K03055), *rpoL* (K03056), *rpoE1* (K03049), and *rpoP* (K03059). Same criteria were used to reconstruct the tree which detailed described in Supplementary Methods. Solid and hollow circles on the branches indicate the bootstrap value ≥ 90 and > 70 respectively.

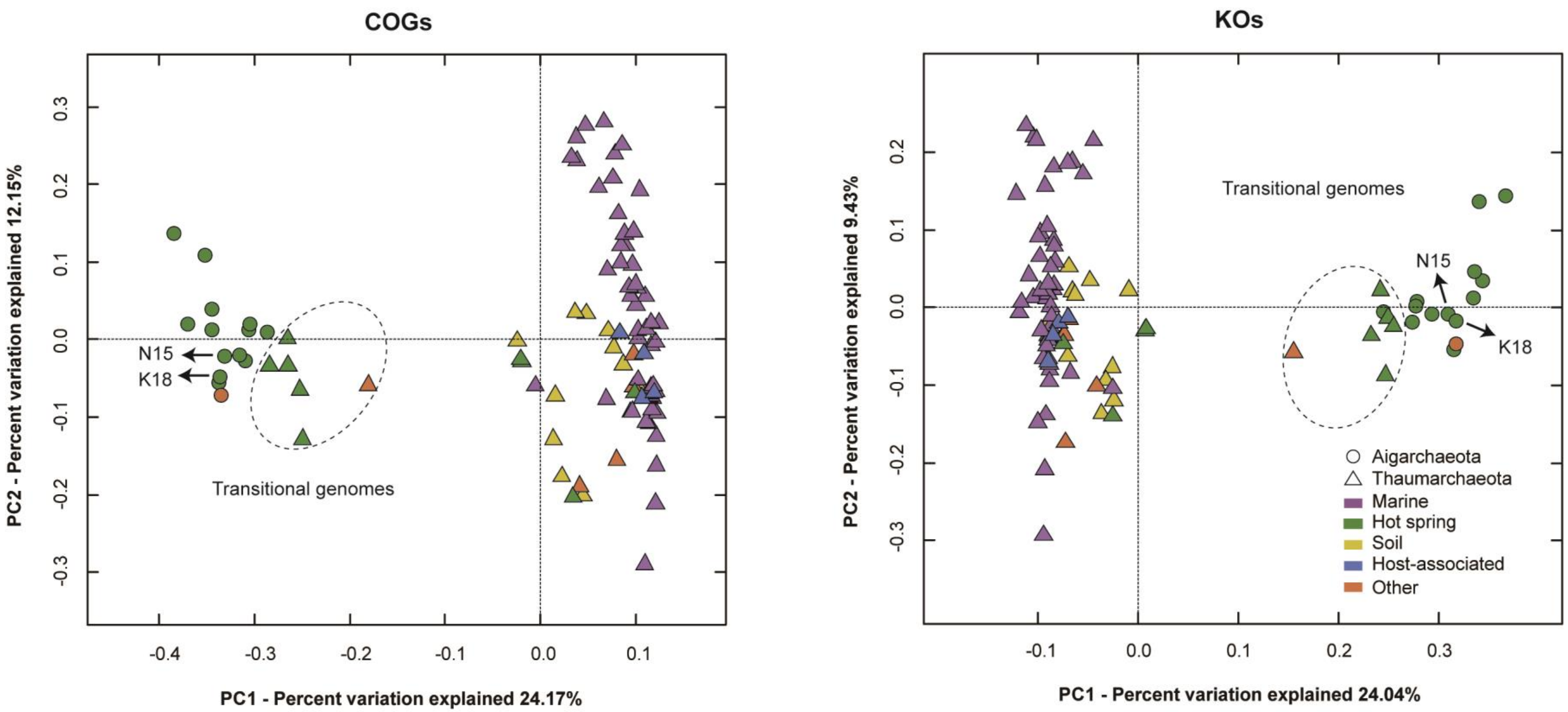

Supplementary Figure 16. Principal coordination analysis (PCoA) plot with Bray-Curtis dissimilarity based on the functional profiling of the 92 genomes (as listed in Table 1; Supplementary Table 4) annotated by (a) KEGG and (b) nogSTRING databases. Genomes in eclipse represent the transitional genomes that show higher functional similarity to Aigarchaeota, but were phylogenetically classified as Thaumarchaeota. N15 and K18 are the abbreviations of strains Aigarchaeota archaeon JGI MDM2 JNZ-1-N15 and Aigarchaeota archaeon JGI MDM2 JNZ-1-K18.

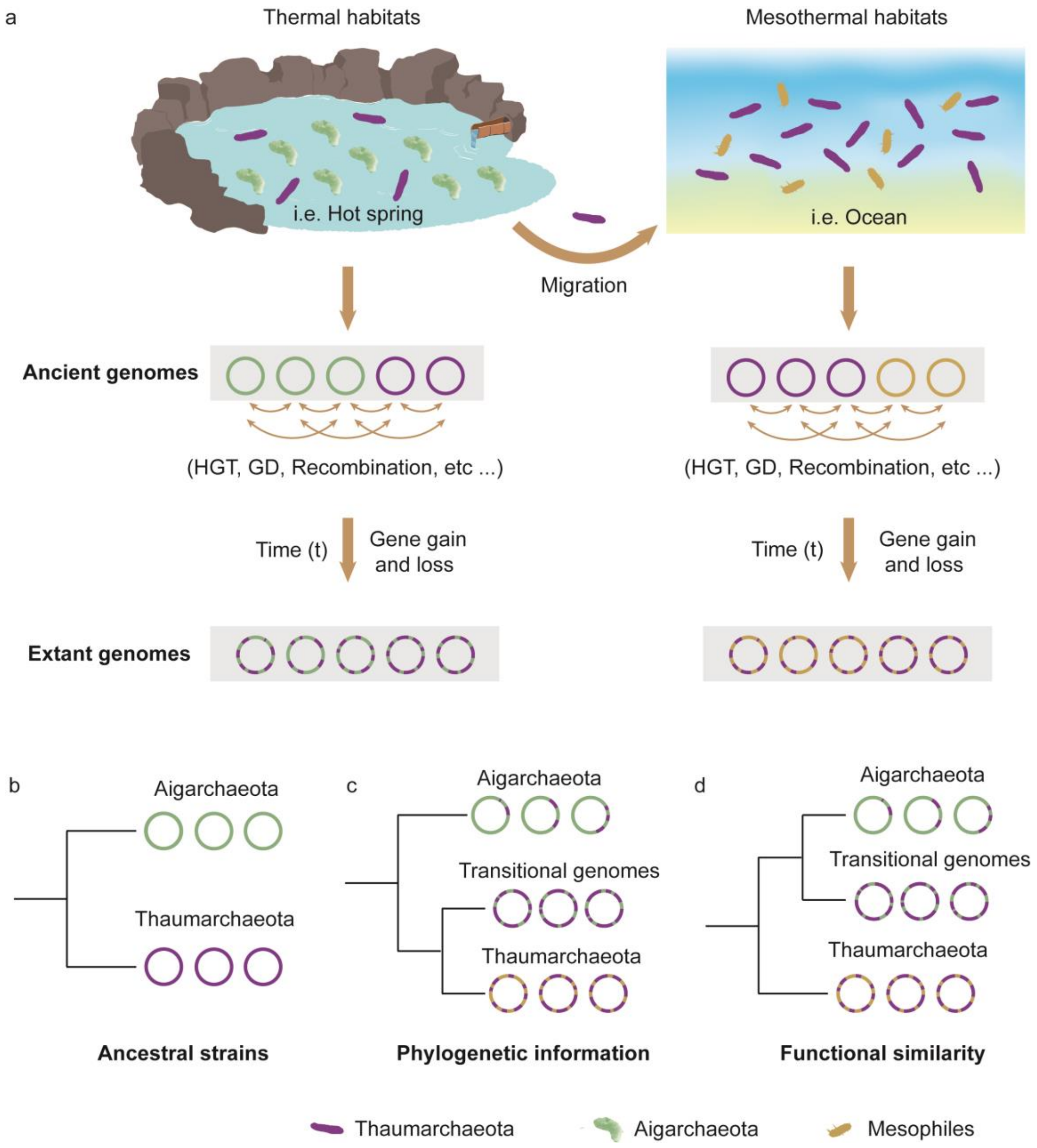

Supplementary Figure 17. (a) The underlying model of the evolutionary history for Aigarchaeota and Thaumarchaeota; (b) The phylogeny of the ancestral strains of Aigarchaeota and Thaumarchaeota; (c) The tree shows the phylogeny of extant genomes of the two phyla; (d) The topology of the clustering based on the functional similarity for the two phyla.

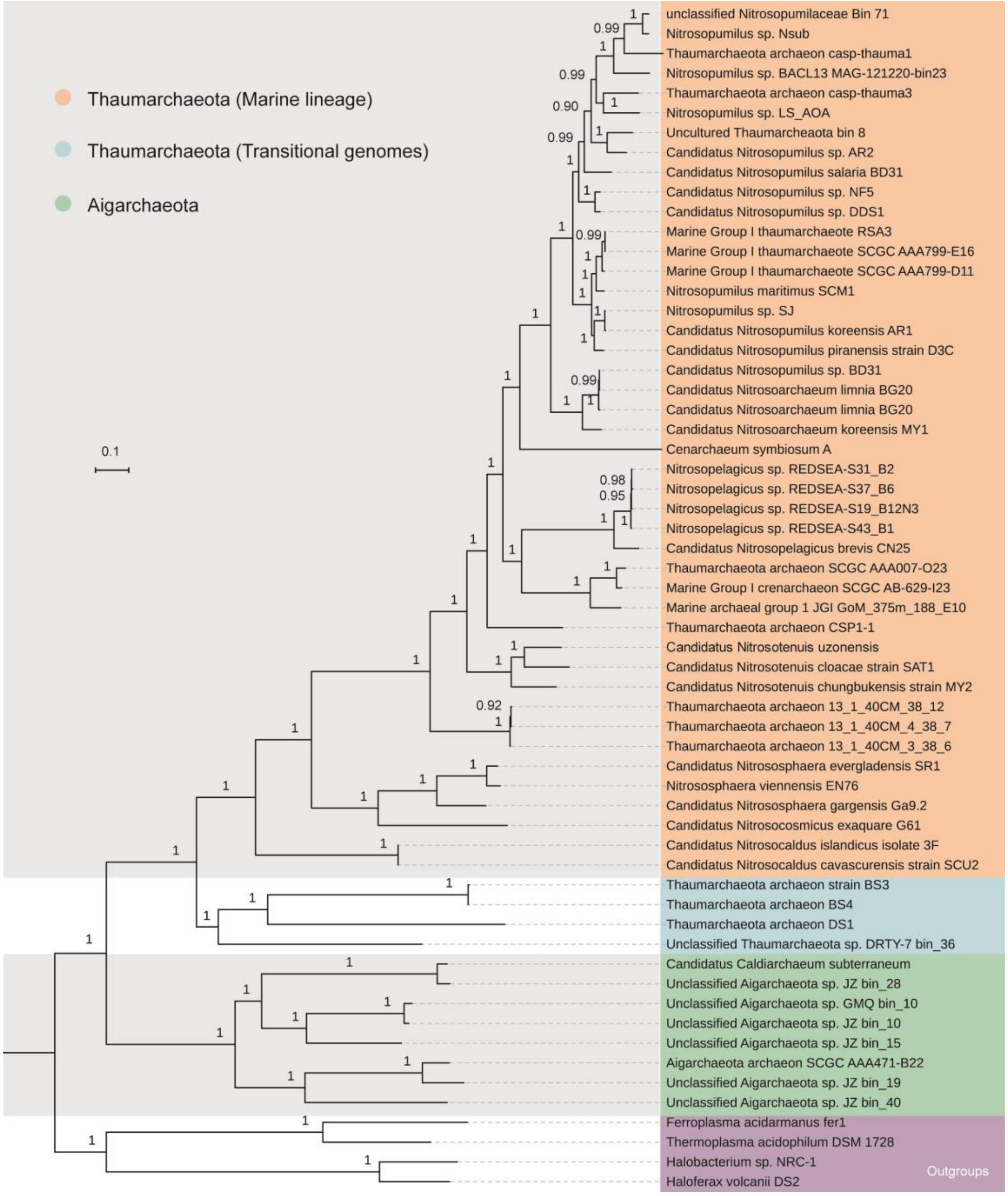

Supplementary Figure 18. The Bayesian tree of Thaumarchaeota and Aigarchaeota reconstructed based on a concatenation of 16 ribosomal protein sequences. Numbers show the posterior possibilities for each node. Four genomes from Euryarchaeota are chosen as outgroups.

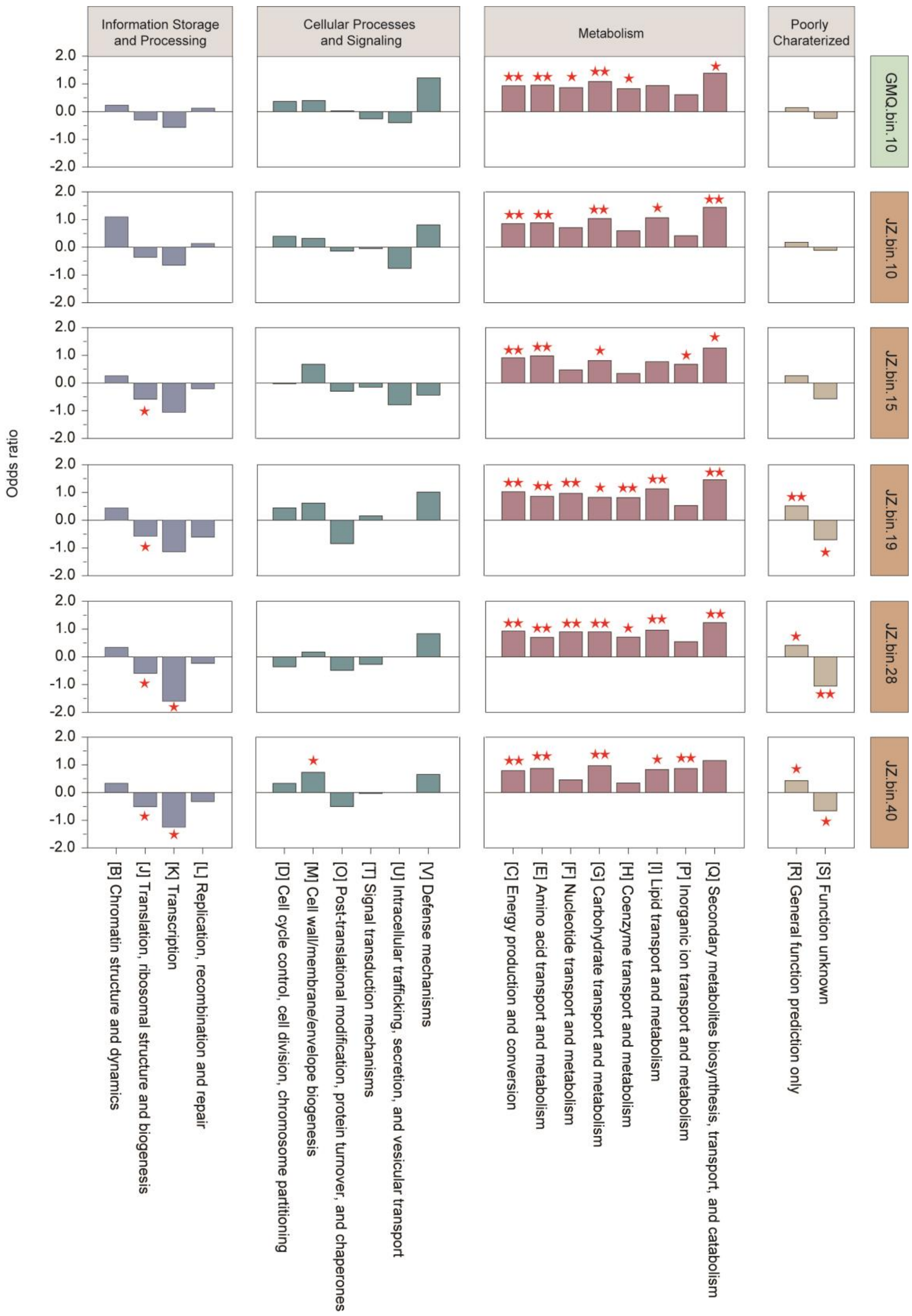

Supplementary Figure 19. Odd ratios of the horizontally transferred genes compared to their whole genomes for the genes annotated as COG functional categories. Asterisks indicate the significant deviation from the null hypothesis (Two-tailed Fisher exact test; * $P < 0.05$; ** $P < 0.01$, $\alpha = 0.01$; P values were adjusted using the “BH” criteria).

Supplementary Tables

Supplementary Table 1. Physical and chemical characteristics of the two hot spring sampling sites.

Sample	Temp.	pH	TOC	DOC	SO_4^{2-}	NH_4^+	NO_3^-	NO_2^-	Na^+	K^+	Mg^{2+}	Ca^{2+}	Cl^-
JZ	75	6.5	6.8	22	19	3.4	3.5	21	300	30	4.0	17	141
GMQ	89	9	0.33	39	41	6.6	3.9	25	772	71	0.50	n.a.	655

All values are in mg L^{-1}, except pH (in standard units), Temperature (in °C) and TOC (in mg g^{-1}).

Abbreviations: TOC, total organic carbon; DOC, dissolved organic carbon; n.a., no detection.

Supplementary Table 2. Carbohydrate degradation related genes identified by comparing to CAZy database.

Gene	GH*	Length	E value	Putative substrates	Description
2718332460	GH109	332	1.3E-17	N-acetylgalactosamine linkage in glycoproteins	UDP-N-acetylglucosamine 3-dehydrogenase
2718332683	GH109	425	1.5E-06	N-acetylgalactosamine linkage in glycoproteins	Predicted dehydrogenase
2718332818	GH109	345	1.7E-18	N-acetylgalactosamine linkage in glycoproteins	Predicted dehydrogenase
2718332892	GH33	769	2.3E-08	Neuraminic acids, glycosidic linkages of terminal sialic acid residues in oligosaccharides	Exo-a-sialidase, neuraminidase
2718332935	GH93	769	2.0E-10	Hemicellulose e.g. a-L-arabinofuranosides, a-L-arabinans containing (1,3)- and/or (1,5)-linkages, arabinoxylans and arabinogalactans	Alpha-L-arabinofuranosidase
	GH51	399	1.4E-93		
2718332966	GH1	490	5.3E-97	Cellulose/hemicellulose	Beta-galactosidase
2718333275	GH36	701	1.0E-236	Terminal a-galactosyl moieties from glycolipids and glycoproteins	Alpha-galactosidase
2718333296	GH109	379	1.8E-08	N-acetylgalactosamine linkage in glycoproteins	Predicted dehydrogenase
2718333341	GH109	336	4.1E-18	N-acetylgalactosamine linkage in glycoproteins	UDP-N-acetylglucosamine 3-dehydrogenase
2718333359	GH31	830	2.0E-174	Starch and disaccharides	Alpha-glucosidase
2718333362	GH78	883	3.0E-188	Pectin	Alpha-L-rhamnosidase
2718333371	GH2	909	6.8E-96	Hemicellulose, b-D-galactoside, b-D-glucuronic acid	Beta-galactosidase/beta-glucuronidase
2718333382	GH29	676	2.3E-19	Hemicellulose	Beta-galactosidase trimerisation domain-containing protein
2718333519	GH109	332	1.5E-18	N-acetylgalactosamine linkage in glycoproteins	Myo-inositol 2-dehydrogenase/D-chiro-inositol 1-dehydrogenase/UDP-N-acetylglucosamine 3-dehydrogenase
2718333575	GH109	333	1.1E-20	N-acetylgalactosamine linkage in glycoproteins	Predicted dehydrogenase
2718333591	GH109	364	4.4E-17	N-acetylgalactosamine linkage in glycoproteins	Predicted dehydrogenase
2718333593	GH109	335	3.4E-07	N-acetylgalactosamine linkage in glycoproteins	Predicted dehydrogenase
2718333594	GH109	343	2.3E-16	N-acetylgalactosamine linkage in glycoproteins	Predicted dehydrogenase
2718333656	GH1	502	3.5E-108	Cellulose/hemicellulose	Beta-glucosidase
2718333662	GH109	358	3.0E-20	N-acetylgalactosamine linkage in glycoproteins	Predicted dehydrogenase
2718333684	GH57	514	2.9E-65	Starch	Alpha-amylase
2718333771	GH109	350	8.8E-14	N-acetylgalactosamine linkage in glycoproteins	Predicted dehydrogenase
2718333842	GH109	377	2.0E-09	N-acetylgalactosamine linkage in glycoproteins	Predicted dehydrogenase
2718333843	GH109	341	2.3E-18	N-acetylgalactosamine linkage in glycoproteins	Predicted dehydrogenase
2718333848	GH38	853	2.7E-80	Mannose	Alpha-mannosidase
2718333904	GH4	462	2.4E-55	Melibiose	Alpha-galactosidase
2718334024	GH127	647	6.1E-193	Release of L-arabinose from specific disaccharides and glycoconjugates	Non-reducing end beta-L-arabinofuranosidase
2718334053	GH109	355	3.7E-19	N-acetylgalactosamine linkage in glycoproteins	Predicted dehydrogenase
2718334057	GH63	663	1.4E-29	Glycogen	Glycogen debranching enzyme
2718334062				Cellulose	Endoglucanase

* Abbreviation: GH, glycosyl hydrolase. GHs are predicted by the comparison to CAZy database[7].

Supplementary Table 3. Distribution of eukaryotic/archaea-specific ribosomal proteins in six aigarchaeal genomes.

KO	Function	GMQ bin_10	JZ bin_10	JZ bin_15	JZ bin_19	JZ bin_28	JZ bin_40	Eukaryotes
K02866	large subunit ribosomal protein L10e	●	●	●	●	●	●	★
K02877	large subunit ribosomal protein L15e	●	●	●	●	●	●	★
K02883	large subunit ribosomal protein L18e	●	●	●	●	●	●	★
K02885	large subunit ribosomal protein L19e	●	●	●	●	●	●	★
K02889	large subunit ribosomal protein L21e	●	●	●	●	●	●	★
K02896	large subunit ribosomal protein L24e	●	●	●	●	●	●	★
K02910	large subunit ribosomal protein L31e	●	●	●	●	●	●	★
K02912	large subunit ribosomal protein L32e	●	●	●	●	●	●	★
K02921	large subunit ribosomal protein L37Ae			●	●	●	●	★
K02922	large subunit ribosomal protein L37e	●	●	●	●	●		★
K02924	large subunit ribosomal protein L39e	●	●	●			●	★
K02929	large subunit ribosomal protein L44e	●	●	●	●	●	●	★
K02930	large subunit ribosomal protein L4e	●	●	●	●	●	●	★
K02936	large subunit ribosomal protein L7Ae	●	●	●	●	●	●	★
K02934	large subunit ribosomal protein L6e							★
K02873	large subunit ribosomal protein L13e			●	●			★
K02875	large subunit ribosomal protein L14e	●	●	●	●	●	●	★
K02882	large subunit ribosomal protein L18ae							★
K02891	large subunit ribosomal protein L22e							★
K02901	large subunit ribosomal protein L27e							★
K02903	large subunit ribosomal protein L28e							★
K02905	large subunit ribosomal protein L29e							★
K02908	large subunit ribosomal protein L30e	●	●	●	●	●	●	★
K02915	large subunit ribosomal protein L34e	●	●	●	●			★
K02917	large subunit ribosomal protein L35ae							★
K02920	large subunit ribosomal protein L36e							★
K02923	large subunit ribosomal protein L38e							★
K02927	large subunit ribosomal protein L40e	●	●	●	●	●	●	★
K02928	large subunit ribosomal protein L41e							★
K02977	small subunit ribosomal protein S27Ae			●	●	●		★
K02984	small subunit ribosomal protein S3Ae	●	●	●	●	●	●	★
K02991	small subunit ribosomal protein S6e	●	●	●	●	●	●	★
K02995	small subunit ribosomal protein S8e	●	●	●	●		●	★
K02987	small subunit ribosomal protein S4e	●	●	●	●	●	●	★
K02993	small subunit ribosomal protein S7e							★
K02962	small subunit ribosomal protein S17e	●	●	●	●			★
K02966	small subunit ribosomal protein S19e	●	●	●	●	●	●	★
K02971	small subunit ribosomal protein S21e							★
K02974	small subunit ribosomal protein S24e	●		●	●	●		★
K02975	small subunit ribosomal protein S25e	●	●	●	●	●	●	★
K02976	small subunit ribosomal protein S26e	●	●	●	●	●	●	★
K02978	small subunit ribosomal protein S27e	●	●	●	●	●	●	★
K02979	small subunit ribosomal protein S28e	●	●	●	●	●	●	★
K02983	small subunit ribosomal protein S30e	●	●	●	●	●		★

Items in grey are ribosomal proteins that are not present in the bacterial and archaeal ribosomes based on a previous study[35].

Supplementary Table 4. Statistics of genetic variations and horizontally transferred genes detected in the six aigarchaeal genomes.

Bins	GMQ.bins.10	JZ.bins.10	JZ.bins.15	JZ.bins.19	JZ.bins.28	JZ.bins.40
Genome size (bp)	1,230,238	1,086,093	1,473,345	1,654,953	1,440,436	1,471,612
No. of protein coding genes	1,398	1,222	1,524	1,734	1,581	1,566
Coding density (%)	90.0	92.5	90.0	89.9	94.7	92.7
Identified HGTs *	278	274	392	372	377	375
Energy production and conversion (%)	35 (12.6)	34 (12.4)	80 (20.4)	57 (15.3)	69 (18.3)	54 (14.4)
Amino acid transport and metabolism (%)	45 (16.1)	48 (17.5)	92 (23.5)	66 (17.7)	62 (16.4)	73 (19.5)
Nucleotide transport and metabolism (%)	16 (5.8)	15 (5.5)	15 (3.8)	32 (8.6)	23 (6.1)	12 (3.2)
Carbohydrate transport and metabolism (%)	29 (10.4)	31 (11.3)	24 (6.1)	19 (5.1)	30 (8)	49 (13.1)
Coenzyme transport and metabolism (%)	20 (7.2)	17 (6.2)	21 (5.4)	27 (7.3)	23 (6.1)	17 (4.5)
Lipid transport and metabolism (%)	8 (2.9)	12 (4.4)	12 (3.1)	32 (8.6)	20 (5.3)	16 (4.3)
Inorganic ion transport and metabolism (%)	15 (5.4)	16 (5.8)	32 (8.2)	22 (5.9)	22 (5.8)	34 (9.1)
Secondary metabolites biosynthesis, transport, and catabol	7 (2.5)	11 (4)	8 (2)	16 (4.3)	12 (3.2)	6 (1.6)
SNPs						
No. of SNPs (%)	2,386	23,321	4,762	5,432	3,172	4,225
Density (%)	0.19	2.14	0.32	0.33	0.22	0.29
Intergenic (%)	344 (14.4)	1698 (7.3)	754 (15.8)	787 (14.5)	250 (7.9)	486 (11.5)
Intragenic (%)	2042 (85.6)	21623 (92.7)	4008 (84.2)	4645 (85.5)	2922 (92.1)	3739 (88.5)
Synonymous (%)	1200 (50.3)	15759 (67.6)	2291 (48.1)	2988 (55.0)	2101 (66.2)	2352 (55.7)
Non-synonymous (%)	842 (35.3)	5864 (25.1)	1717 (36.1)	1657 (30.5)	821 (25.9)	1387 (32.8)
Indels						
No. of indels	98	295	173	82	34	74
Percentage (PPM)	80	272	117	50	24	50

* HGTs were indentified using HGTector (Zhu et al., 2014) as detailed described in Methods.

Abbreviations: HGT, horizontal gene transfer; SNP, single nucleotide polymorphism; PPM, percentage per million.

Supplementary References

1. Hyatt, D. et al. Prodigal: prokaryotic gene recognition and translation initiation site identification. BMC *Bioinformatics* **11**, 119 (2010).
2. Lagesen, K. et al. RNAmmer: consistent and rapid annotation of ribosomal RNA genes. *Nucleic Acids Res.* **35**, 3100-3108 (2007).
3. Kanehisa, Furumichi, M., Tanabe, M., Sato, Y., & Morishima, K., KEGG: new perspectives on genomes, pathways, diseases and drugs. *Nucleic Acids Res.* **45**, D353-D361 (2017)
4. Makarova, K. S., Wolf, Y. I., & Koonin, E. V. Archaeal clusters of orthologous genes (arCOGs): an update and application for analysis of shared features between thermococcales, methanococcales, and methanobacteriales. *Life* **5**, 818-840 (2015).
5. Huerta-Cepas, J. et al. eggNOG 4.5: a hierarchical orthology framework with improved functional annotations for eukaryotic, prokaryotic and viral sequences. *Nucleic Acids Res.* **44**, D286-D293 (2016).
6. Buchfink, B., Xie, C. & Huson, D. H. Fast and sensitive protein alignment using DIAMOND. *Nat. Methods* **12**, 59-60 (2015).
7. Cantarel, B. L., Coutinho, P. M., Rancurel, C., Bernard, T., Lombard, V., & Henrissat, B. The Carbohydrate-Active EnZymes database (CAZy): an expert resource for glycogenomics. *Nucleic Acids Res.* **37**, D233-D238 (2008).
8. Marchler-Bauer, A. et al. CDD: NCBI's conserved domain database. *Nucleic Acids Res.* **43**, D222-D226 (2014).
9. Moriya, Y., Itoh, M., Okuda, S., Yoshizawa, A. C. & Kanehisa, M. KAAS: an automatic genome annotation and pathway reconstruction server. *Nucleic Acids Res.* **35**, 182-185 (2007).
10. Grissa, I., Vergnaud, G. & Pourcel, C. CRISPRFinder: a web tool to identify clustered regularly interspaced short palindromic repeats. *Nucleic Acids Res.* **35**, 52-57 (2007).
11. Marreiros, B. C., Batista, A. P., Duarte, A. M., & Pereira, M. M. A missing link between complex I and group 4 membrane-bound [NiFe] hydrogenases. *Biochim. Biophys. Acta* **1827**, 198-209 (2013).
12. De Castro, E. et al. ScanProsite: detection of PROSITE signature matches and ProRule-associated functional and structural residues in proteins. *Nucleic Acids Res.* **34**, W362-W365 (2006).
13. Jones, P. et al. InterProScan 5: genome-scale protein function classification. *Bioinformatics* **30**, 1236-1240 (2014).
14. Biasini, M. et al. SWISS-MODEL: modelling protein tertiary and quaternary structure using evolutionary information. *Nucleic Acids Res*. **42**, W252-W258 (2014).
15. Hug, L. A. et al. A new view of the tree of life. *Nat. Microbiol*. **1**, 16048 (2016).
16. Wu, M. & Scott, A. J. Phylogenomic analysis of bacterial and archaeal sequences with AMPHORA2. *Bioinformatics* **28**, 1033-1034 (2012).
17. Edgar, R. C. MUSCLE: multiple sequence alignment with high accuracy and high throughput. *Nucleic Acids Res.* **32**, 1792-1797 (2004).
18. Capella-Gutiérrez, S., Silla-Martínez, J. M. & Gabaldón, T. trimAl: a tool for automated alignment trimming in large-scale phylogenetic analyses. *Bioinformatics* **25**, 1972-1973 (2009).
19. Stamatakis, A. RAxML-VI-HPC: maximum likelihood-based phylogenetic analyses with thousands of taxa and mixed models. *Bioinformatics* **22**, 2688-2690 (2006).
20. Cole, J. R. et al. Ribosomal Database Project: data and tools for high throughput rRNA analysis. *Nucleic Acids Res.* gkt1244 (2013).
21. Pruesse, E. et al. SILVA: a comprehensive online resource for quality checked and aligned ribosomal RNA sequence data compatible with ARB. *Nucleic Acids Res.* **35**, 7188-7196 (2007).
22. Pruesse, E., Peplies, J. & Glöckner, F. O. SINA: accurate high-throughput multiple sequence alignment of ribosomal RNA genes. *Bioinformatics* **28**, 1823-1829 (2012).
23. Letunic, I. & Bork, P. Interactive tree of life (iTOL) v3: an online tool for the display and annotation of phylogenetic and other trees. *Nucleic Acids Res.* **44**, 242-245 (2016).
24. Müller, A. L., Kjeldsen, K. U., Rattei, T., Pester, M., & Loy, A. Phylogenetic and environmental diversity of DsrAB-type dissimilatory (bi) sulfite reductases. *ISME J.* **9**, 1152-1165. (2015).
25. Li, W., & Godzik, A. Cd-hit: a fast program for clustering and comparing large sets of protein or nucleotide sequences. *Bioinformatics* **22**, 1658-1659 (2006).
26. King, G. A. Molecular and culture-based analyses of aerobic carbon monoxide oxidizer diversity. *Appl. Environ. Microbiol*. **69**, 7257-7265 (2003).
27. King, G. M., & Weber, C. F. Distribution, diversity and ecology of aerobic CO-oxidizing bacteria. *Nat. Rev. Microbiol.* **5**, 107-118. (2007).
28. Greening, C. et al. Genomic and metagenomic surveys of hydrogenase distribution indicate H2 is a widely utilised energy source for microbial growth and survival. *ISME J.* **10**, 761-777. (2015).

29. Thompson, J. D., Gibson, T., & Higgins, D. G. Multiple sequence alignment using ClustalW and ClustalX. *Curr Protoc Bioinformatics*, 2-3 (2002).
30. Tamura, K., Stecher, G., Peterson, D., Filipski, A., & Kumar, S. MEGA6: molecular evolutionary genetics analysis version 6.0. *Mol. Biol. Evol.* **30**, 2725-2729 (2013).
31. Dick, G. J. et al. Community-wide analysis of microbial genome sequence signatures. *Genome Biol*. **10**, R85 (2009).
32. Frazer, K. A., Pachter, L., Poliakov, A., Rubin, E. M., & Dubchak, I. VISTA: computational tools for comparative genomics. *Nucleic Acids Res.* **32**, W273-W279. (2004).
33. Waterhouse, A. M., Procter, J. B., Martin, D. M., Clamp, M., & Barton, G. J. Jalview Version 2—a multiple sequence alignment editor and analysis workbench. *Bioinformatics* **25**, 1189-1191 (2009).
34. Marchler-Bauer A. et al. CDD/SPARCLE: functional classification of proteins via subfamily domain architectures. *Nucleic Acids Res.* **45**, 200-203 (2017).
35. Armache, J. P. et al. Localization of eukaryote-specific ribosomal proteins in a 5.5-Å cryo-EM map of the 80S eukaryotic ribosome. *Proc. Natl Acad. Sci. USA.* **107**, 19754-19759 (2010).
