# Supplementary material for: Genomic inference of the metabolism and evolution of the archaeal phylum Aigarchaeota
Source: Nat Commun. 2018 Jul 19;9:2832. doi: 10.1038/s41467-018-05284-4 (PMC6053391; doi:10.1038/s41467-018-05284-4)
Supplement: Supplementary file 3 — Description of Additional Supplementary Files [file 41467_2018_5284_MOESM3_ESM.pdf]

Description of Additional Supplementary Files

File Name: Supplementary Data 1
Description: List of genes and featured in Figure 2 in the main text.

File Name: Supplementary Data 2
Description: The genome characteristics of the selected 87 draft genomes (downloaded from public databases) in this research.

File Name: Supplementary Data 3
Description: Detected gene gain and loss events at key nodes.

File Name: Supplementary Data 4
Description: Identified potential horizontal transferred genes for the six aigarchaeal bins.
